# Between life and death: the brain twilight zones

**DOI:** 10.3389/fnins.2023.1156368

**Published:** 2023-05-15

**Authors:** Stéphane Charpier

**Affiliations:** ^1^Sorbonne Université, Institut du Cerveau – Paris Brain Institute - ICM, Inserm, CNRS, APHP, Hôpital de la Pitié-Salpêtriére, Paris, France; ^2^Sorbonne University, UPMC Université Paris, Paris, France

**Keywords:** cortex, anoxia, excitability, intracellular record, death, resuscitation

## Abstract

Clinically, and legally, death is considered a well-defined state of the organism characterized, at least, by a complete and irreversible cessation of brain activities and functions. According to this pragmatic approach, the moment of death is implicitly represented by a discrete event from which all cerebral processes abruptly cease. However, a growing body of experimental and clinical evidence has demonstrated that cardiorespiratory failure, the leading cause of death, causes complex time-dependent changes in neuronal activity that can lead to death but also be reversed with successful resuscitation. This review synthesizes our current knowledge of the succeeding alterations in brain activities that accompany the dying and resuscitation processes. The anoxia-dependent brain defects that usher in a process of potential death successively include: (1) a set of changes in electroencephalographic (EEG) and neuronal activities, (2) a cessation of brain spontaneous electrical activity (isoelectric state), (3) a loss of consciousness whose timing in relation to EEG changes remains unclear, (4) an increase in brain resistivity, caused by neuronal swelling, concomitant with the occurrence of an EEG deviation reflecting the neuronal anoxic insult (the so-called “wave of death,” or “terminal spreading depolarization”), followed by, (5) a terminal isoelectric brain state leading to death. However, a timely restoration of brain oxygen supply—or cerebral blood flow—can initiate a mirrored sequence of events: a repolarization of neurons followed by a re-emergence of neuronal, synaptic, and EEG activities from the electrocerebral silence. Accordingly, a recent study has revealed a new death-related brain wave: the “wave of resuscitation,” which is a marker of the collective recovery of electrical properties of neurons at the beginning of the brain’s reoxygenation phase. The slow process of dying still represents a terra incognita, during which neurons and neural networks evolve in uncertain states that remain to be fully understood. As current event-based models of death have become neurophysiologically inadequate, I propose a new mixed (event-process) model of death and resuscitation. It is based on a detailed description of the different phases that succeed each other in a dying brain, which are generally described separately and without mechanistic linkage, in order to integrate them into a continuum of declining brain activity. The model incorporates cerebral twilight zones (with still unknown neuronal and synaptic processes) punctuated by two characteristic cortical waves providing real-time biomarkers of death- and resuscitation.

*“Death is not a life event. It is not a fact of the world”*

L. Wittgenstein. Notebooks 1914-1916

## 1. Introduction

### 1.1. The “event models” of death

In our modern societies, regardless of spiritual or religious beliefs, death is considered from two drastically different points of view (Morison, 1971; Azevedo and Bitencourt Othero, 2020). On the one hand, it is regarded as a novel physical configuration of the organism that settles instantly, and definitively, after a clearly defined event (dying), which marks the complete and definitive failure of integrative physiological processes. On the other, dying is no more a single, momentary, phenomenon but a long-drawn-out process that is established from the beginning of life and completed when all cells have stopped converting energy. This overstated vision reflects an erroneous interpretation of the neovitalism advocated by the nineteenth-century French physiologist Xavier Bichat, who affirmed that “…*life is the set of functions that resist death*” (Bichat, 1800). This misunderstanding is the origin of fundamental incoherence because it would make synonymous dying, aging, and living (Kass, 1971).

**Figure 1:**
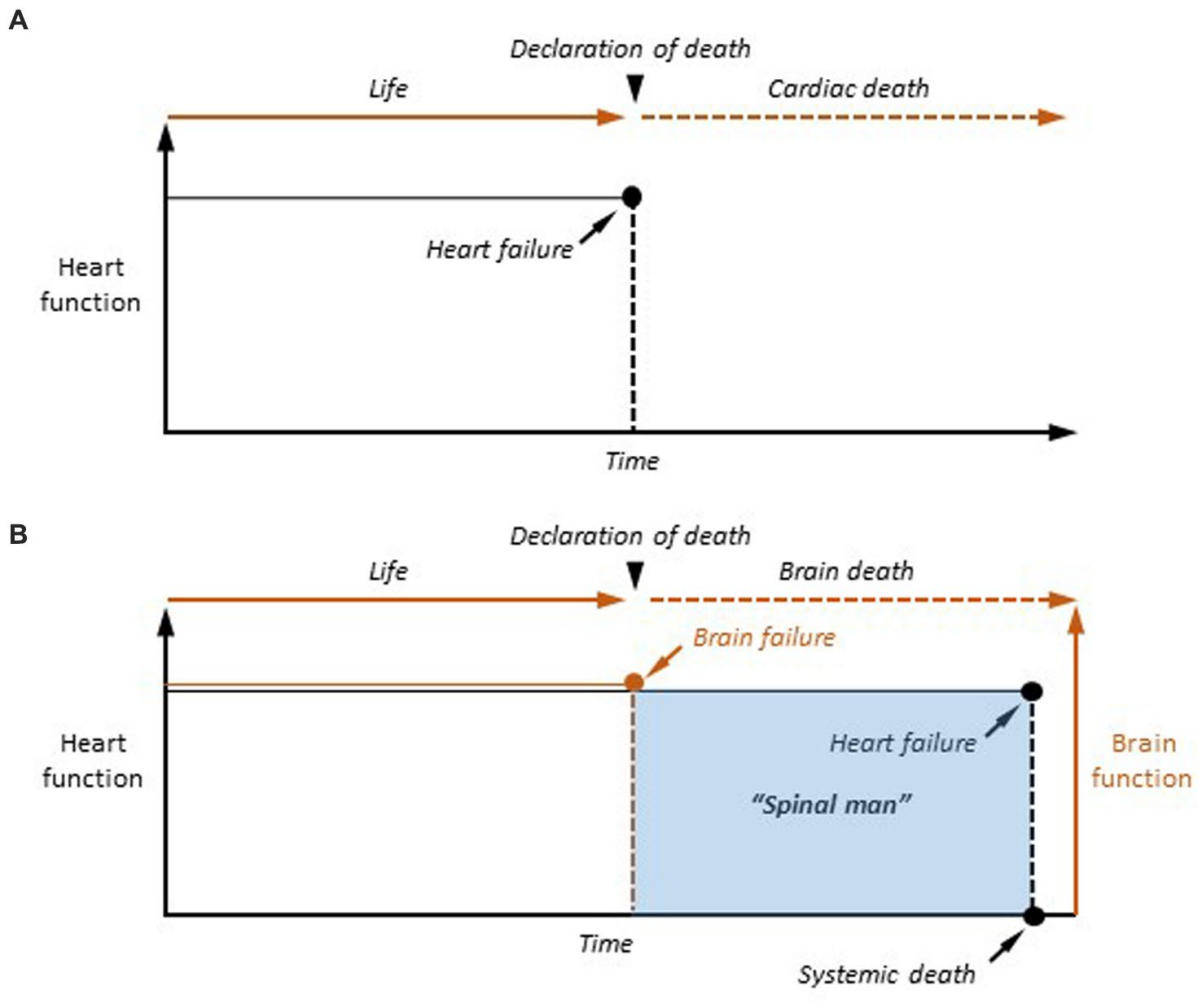
The two event-based models of death. **(A)** Cardiac death. In case of definitive arrest of cardiac function, the moment of death is determined as the last heartbeat. **(B)** Brain death. Once the criteria of cerebral death are clinically established (Brain failure), the patient is considered as dead. However, cardiorespiratory functions are artificially maintained in the brain-dead person, making the human being a “spinal man” (see text for explanation). Systemic death is achieved, after variable delays, after the terminal interruption of respiratory activity, and the subsequent cessation of heartbeat.

The first point of view is certainly the most popular because it complies with the need for concreteness when considering a person living or dead. It is indeed unacceptable, emotionally (when it concerns a relative), clinically and legally, to declare that an individual is “semi-living” or “semi-dead,” or in an unspecified physiological state, stuck between life and death. The all-or-nothing *event model* of death thus prevails as it is both symbolically appropriate and operationally effective when applied to the medical field. Furthermore, it is supposed to be based on unbiased criteria for determining that a death has occurred, and thus to allow for an accurate determination of the time of death, whether it is cardiorespiratory or caused by brain failure, the two currently recognized forms of death (Figure 1).

In the case of cardiac failure, the transition from the living to the dead state is realized, from a temporal and functional point of view, by the last heartbeat, a single event that is sufficient in itself to provoke the cessation of all physiological functions as it causes a rapid collapse of cell metabolism (Figure 1A). Since the description of the *coma depassé* by Mollaret and Goulon (1959) and the recognition of a definitive brain failure as the second form of death by the Harvard committee (A definition of irreversible coma. Report of the *Ad Hoc* Committee of the Harvard Medical School to Examine the Definition of Brain Death, 1968), dying has undergone a paradigm shift, as permanent cardiorespiratory arrest is no longer necessary to be declared dead. Brain death results from catastrophic and irreversible brain injuries causing the fatal quadrilogy of neurological signs, including loss of brainstem reflexes, inability to breathe spontaneously (brain-dead individuals are kept on a ventilator), absence of spontaneous electroencephalographic (EEG) activity (isoelectric state), and dissolution of conscious processes in all their dimensions (coma) (Wijdicks, 2013). The *event model* of death is also applicable to brain death. In this case, the moment of death is the moment when permanent cessation of vital brain functions (apnea) and permanent unconsciousness (coma) are attested (Figure 1B). However, in contrast to cardiac death, brain-dead patients are maintained under artificial respiration, their heart still beats, and the other organs remain viable—as they are perfused by oxygenated arterial blood –, including the spinal cord, making them “spinal men” (Turmel et al., 1991).

Although cardiac and brain deaths are now equivalent in law and medicine, the brain has become the only organ whose definitive functional cessation is sufficient and necessary to consider a human death. The cardio-centered vision of death has thus given way to a cerebro-centered form, whose hallmark is the final loss of consciousness in a heartbeat cadaver (Figure 1B). The consequence is that understanding the transition from life to death requires elucidating the mechanisms underlying the transition from an active brain to an irreversibly altered brain, rendering individuals permanently unconscious.

### 1.2. Arguments in favor of a “process model”

Whether due to cardiorespiratory or brain failure, death is in fact preceded by time-dependent changes in brain activity. The first measurable changes are modifications in the brain’s spontaneous electrical patterns, which slowly evolve in time (over several minutes) until brain activity permanently ceases. These modifications can be readily and non-invasively captured using surface EEG and can also be detected in individual neurons—from the brainstem to neocortical cells –, albeit with great variability in their arrival time from anoxia onset (Peña and Ramirez, 2005; Brisson et al., 2013).

**Figure 2:**
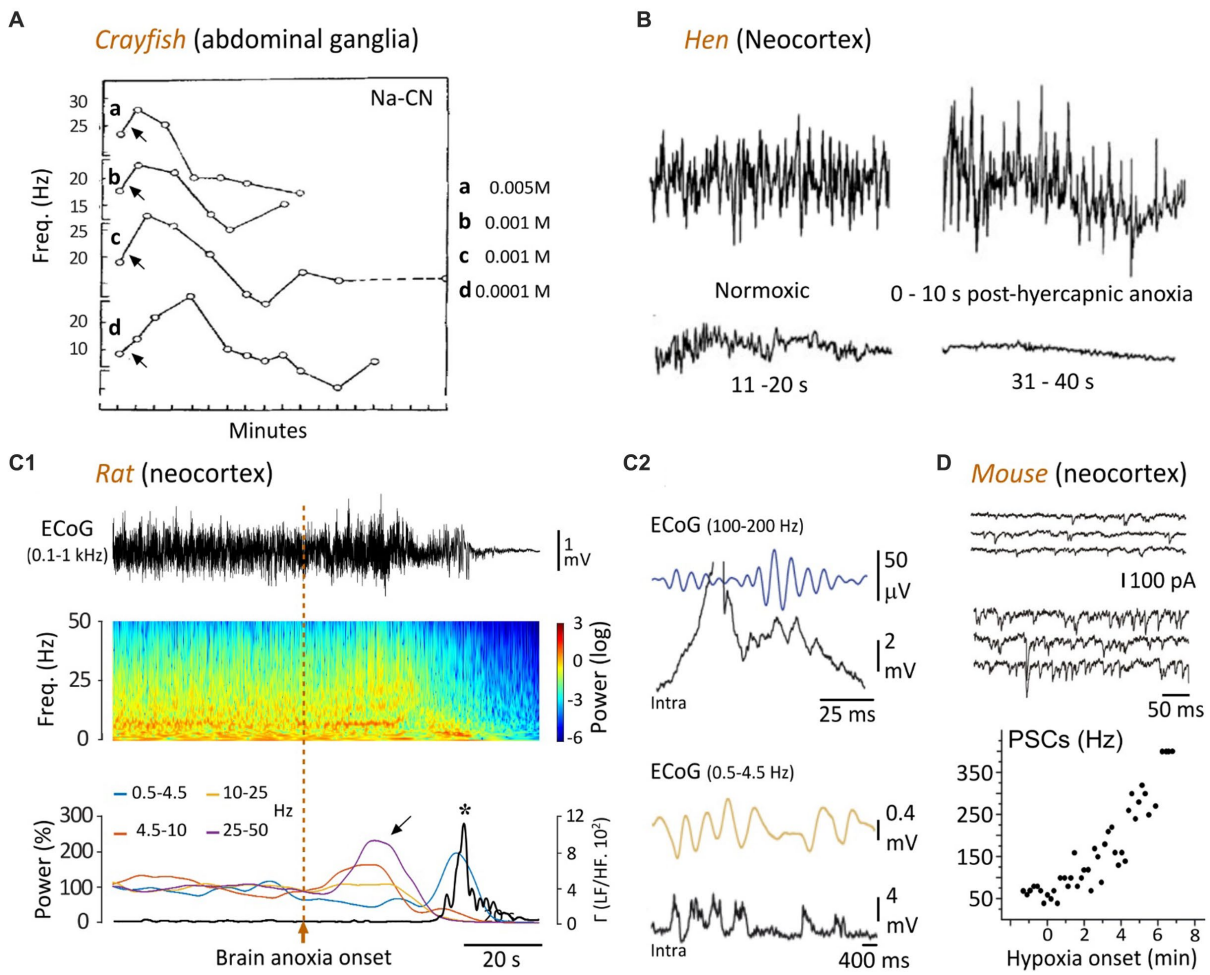
Temporal evolution of spontaneous neuronal activity in response to oxygen deprivation. **(A)** The ongoing firing of crayfish ganglionic neurons is transiently increased by applications (arrows) of cyanide (CN-Na), then declines rapidly. The different concentrations of CN-Na are indicated (modified from Prosser and Buehl, 1939). **(B)** EEG recordings from a hen showing that hypercapnic hypoxia (49% carbon dioxide in air) causes an initial augmentation of the cortical waveforms, followed by a gradual attenuation until reaching an isoelectric state after ~30 s (modified from Mohan Raj et al., 1992). **(C)** Time course of change in cortical activity in the rat upon asphyxiation. **(C1)** ECoG record (top) prior to, and after, anoxia induced by asphyxia (vertical dashed line), and corresponding time-frequency map (middle). The graph below indicates the relative power of the different frequency bands (normalized as a function of their mean control value) over time. Note the surges of high-frequency oscillations during the early anoxic period (oblique arrow), followed by a bump of low frequency activity. The bold line represents the power ratio of low frequencies (LF, 0.5–4.5 Hz) to merged faster components (HF, 10–50 Hz). The peak of LF/HF ratio (asterisk) after anoxia onset corresponds to an augmentation of 9,800% relative to the control value. **(C2)** Intracellular correlates (Intra) of high (top) and low (bottom) frequency ECoG activities, as simultaneously recorded in a subjacent layer 5 pyramidal neuron. The ECoG signals are filtered between 25 and 50 Hz (top) or between 0.5 and 4.5 Hz (bottom), to isolate the two frequency bands (modified from Schramm et al., 2020). **(D)** *In vitro* voltage-clamp recordings of a layer 5 cortical neuron (held at −70 mV), in normoxic condition (top records) and during hypoxia (bottom records). The graph shows the frequency of post-synaptic currents (PSCs) as a function of time, before and during hypoxia, obtained from the same neuron (modified from Revah et al., 2016).

**Figure 3:**
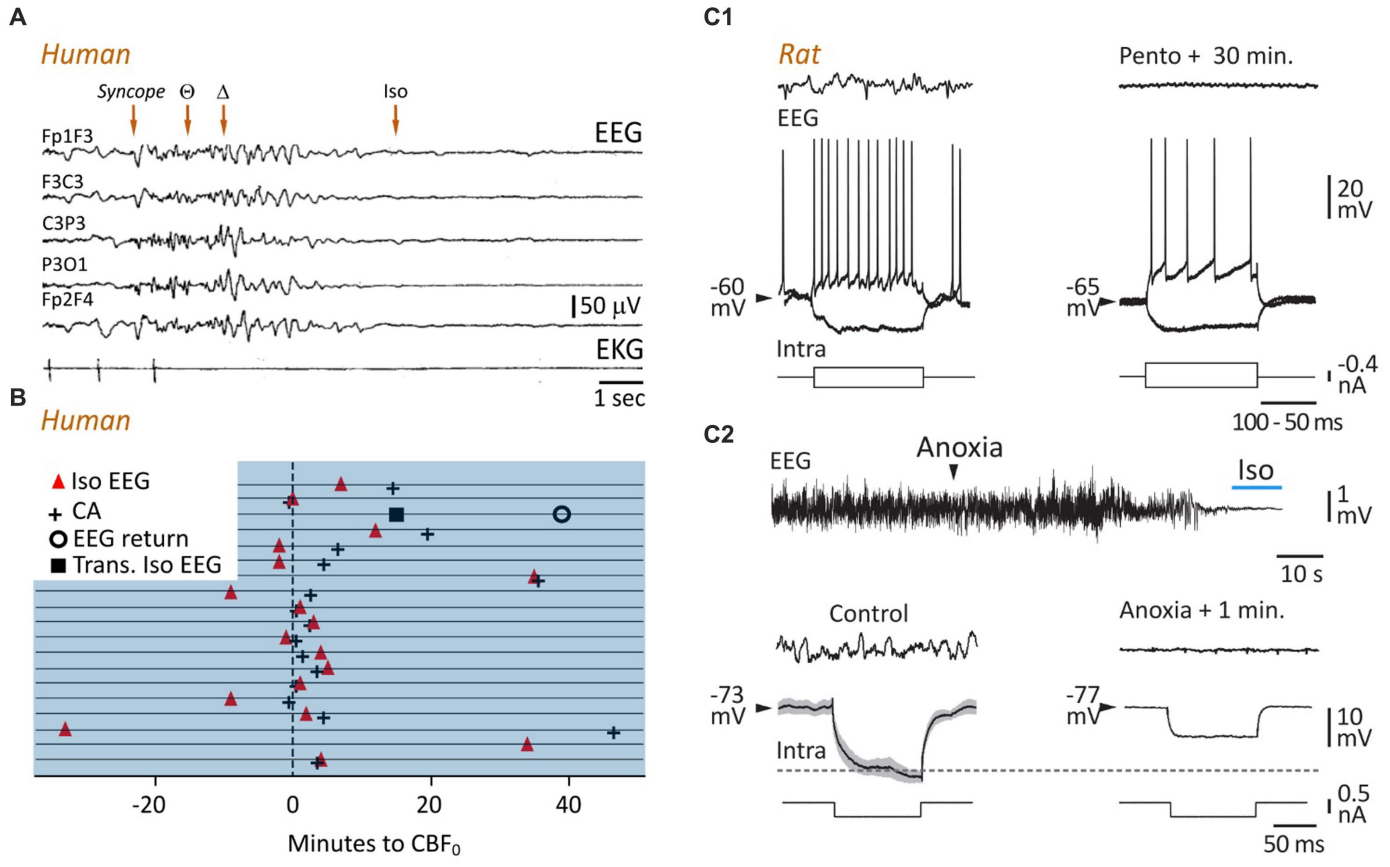
A persisting lack of brain oxygenation causes electrocerebral inactivity. **(A)** Time course of change of EEG during cardioinhibitory vasovagal syncope. In this patient, with a history of recurrent syncope, head-up tilt testing provoked a loss of consciousness (Syncope) that correlated with cardiac arrest (bottom record), rapidly followed by a slowing in EEG waveforms, from theta to delta patterns, which vanish to give way to an isoelectric trace approximately 10 s after the last systole (modified from Ammirati et al., 1998). **(B)** Timings of electrocerebral silence on EEG (Iso EEG) and cardiac arrest (CA) in relation to cessation of cerebral blood flow (CBF_0_). Horizontal lines represent 19 patients, and the *x* axis represents time in minutes. In one patient, the appearance of the isoelectric EEG was transient (Trans. Iso EEG), with a restoration, also momentary, of cortical waveforms approximately 40 s after brain circulation was stopped (modified from Matory et al., 2021). **(C)** Comparison of cellular correlates of isoelectric EEG induced by pharmacological inhibition **(C1)** or brain anoxia **(C2)**, in rats *in vivo*. EEG activities (top records) were captured simultaneously with intracellular recordings of subjacent pyramidal neurons (bottom records). **(C1)** Voltage responses of a neuron to depolarizing and hyperpolarizing currents pulses in control (left) and 30 min. After systemic injection of pentobarbital (Pento) causing isoelectric EEG. Note that the membrane resistance was not affected (negative voltage deflections) and the persistence of the current-evoked firing while the EEG was flat (modified from Altwegg-Boussac et al., 2014) (**C2)**. *Top*: Time course of change of EEG waveforms during asphyxia-induced anoxia. Bottom: Same representation as in **(C1)**. Note that the membrane resistance and time constant of the neuron were reduced after one minute of anoxia as compared to the normoxic period (horizontal dashed line). The measurement was performed at the period indicated on the isoelectric EEG (top, blue line) (modified from Schramm et al., 2020).

Alterations in brain electrical activity before death do exist regardless of the phylogenetic position of the species (from invertebrates to humans) (Spong et al., 2016; Figures 2, 3), with a globally conserved sequence of changes despite a disparate sensitivity to oxygen deprivation from one vertebrate species to another (Nilsson and Lutz, 2004). The first evidence of a change in electrical brain activity before death was obtained by Richard Caton, the discoverer of the EEG (Caton, 1875), who described in various mammalian species an initial “considerable” increase in cortical currents after massive hemorrhage causing death (Caton, 1877, 1887). Given the limited frequency range of the vibrations of the galvanometer used at that time, it is not possible to decide here between a surge of high-frequency neuronal activity and the slow and large-amplitude brain current underlying the neuronal “anoxic depolarization.” Caton also found that brain currents dropped to “zero” once death was definitively established (Caton, 1877, 1887), which probably corresponded to the terminal flat EEG observed in case of cardiac or brain death. This dissolution of cerebral electrical activity during the dying process was further confirmed by Pravdich-Neminsky in 1912, who provides the first pictorial tracing of a slowing EEG in response to cerebral ischemia (Niedermeyer and da Silva, 2005).

In light of recent findings, I will review the sequential electrical and functional changes that occur in the brain after an anoxic insult that can lead to death and discuss their mechanisms. I will focus on the cerebral cortex, whose functional breakdown and correlative loss of consciousness makes it a key structure for the study of death in human. Next, I will propose a new, mixed (event-process), model of death and resuscitation, incorporating cerebral twilight zones, which represent periods during which brain activity progressively declines after global anoxia to an irreversible state, i.e., a point of no return to a state of death, or to a process of recovery after successful resuscitation. In this model, two twilight zones must be distinguished. The first, common to both irreversible and reversible outcomes, lasts until the onset of anoxic depolarizations, which are the cardinal cortical events in a brain undergoing global anoxia. An attempt at resuscitation results in a second twilight zone. From this point on, the brain evolves in an uncertain state, during which a return of cortical activities can be reliably predicted while the time when no return is possible is still unknown.

## 2. Initial cortical changes: from fast to slow activity

The mammalian brain is metabolically expensive, since it represents only a small fraction of total body weight, but accounts for 20–25% of oxygen consumption and glucose utilization. Most of the brain’s energy (75–90%) is used for neuronal signaling processes, primarily at the synapses but also in the homeostatic control of ion gradients by ATP-dependent ion pumps, and action potential generation and propagation (for a detailed overview of neuronal metabolism see Hofmeijer and van Putten, 2012; Watts et al., 2018). As a result, neurons are very sensitive to disruptions in oxygen supply, which causes rapid damage to synapses and cellular excitability, especially during dramatic events that can lead to death by cessation of oxygenated blood perfusion in the brain. Accordingly, our current view of the electrical changes that arise in the brain as death approaches is based on clinical investigations and experiments conducted in various animal models, using global cerebral ischemia (cardiac arrest or basal cerebral artery ligation), inhalation of toxic gasses, drug intoxication, and asphyxia.

The different phases of brain activity decline have been originally described by Baumgartner et al. (1961) utilizing EEG recordings in a cat model of systemic anoxia (breathing 100% nitrogen). They identified four distinct phases of activity changes, including (1) a time interval during which no change in activity is observed, (2) an activation period, within 10–20 s after anoxia onset, during which EEG shows arousal-type patterns (15–40 Hz), (3) a subsequent period of slow oscillations in the delta range (1–5 Hz) and, (4) a terminal isoelectric EEG, initially called the “null phase.” We will see in the next paragraphs that none of these stages in the process of brain activity fading constitutes a point of no return, and, that the initial phase of electrocerebral inactivity can be followed, when anoxia is maintained, by a large-amplitude slow EEG event marking the beginning of the neuronal death process.

### 2.1. Fast activity: from neurons to EEG

A transient increase in neuronal activity in response to metabolic impairment was originally described in the abdominal ganglia of crayfish subjected to various types of asphyxiation. In this invertebrate species, application of nitrogen, sodium cyanide, carbon monoxide, or 2,4-dinitrophenol induced what was called a “stimulation” of the neurons during the first minutes, characterized by an increase by 5–15 Hz of their spontaneous firing frequency (Prosser and Buehl, 1939). This initial augmentation of cell activity was then followed by a gradual decrease until its interruption after 5–10 min (Figure 2A). Similar findings were found in the lobster somatosensory ganglion, in which the duration of cyclic burst firing was transiently increased after lowering of oxygen pressure (Massabuau and Meyrand, 1996). Interestingly, in the crayfish, the delayed carbon monoxide-induced inhibition of neuronal firing could be reversed by brief exposure to light (500 watts), which increases ATP production after its absorption by cytochrome *c* oxidase (Wong-Riley et al., 2005).

In birds (hens) implanted with neocortical electrodes, a severe brain anoxia (air-breathing saturated in argon) resulted in a transient amplification of EEG waveforms ~10 s after oxygen deprivation (Figure 2B), which correlated with a loss of posture (Raj et al., 1991; Mohan Raj et al., 1992). This increase in brain activity was followed by a progressive suppression of EEG signals, a closure of the eyes, a canceling of somatosensory-evoked potentials (~30-s post-anoxia), and finally the occurrence of an isoelectric EEG about 1 min after anoxia onset (Raj et al., 1991; Mohan Raj et al., 1992; Figure 2B). Remarkably, as in humans (see below), the apparent loss of consciousness (disintegration of the waking behavioral appearance) seems to occur before the establishment of electrocerebral inactivity.

The rodent models have allowed decisive advances in the understanding of cortical dynamic changes that rapidly follow a lack of brain oxygenation or complete cerebral ischemia. They confirmed and extended the previous findings obtained from invertebrates, birds, cats, and those initially identified in humans. In the rat, cardiac arrest, induced by intra-cardiac injection of potassium chloride (Borjigin et al., 2013), decapitation (Mikeska and Klemm, 1975; Vanderwolf et al., 1988; Kongara et al., 2014), injection of a lethal dose of anesthetics (Zhang et al., 2019) or interruption of breathing in paralyzed animals (Lee et al., 2017; Schramm et al., 2020), lead to a surge of high-frequency electrical activity in the neocortex within the first 40 s preceding the isoelectric line. A detailed analysis of cortical activity dynamics in the cardiac arrest (Borjigin et al., 2013) and asphyxia (Lee et al., 2017; Schramm et al., 2020) models indicated complex time-dependent changes, including a primary diffuse increase in a wide range of gamma frequencies (25–130 Hz), within 10–35 s after anoxia onset (Figure 2C1), accompanied by a rise in frontal coherence between the left and right motor cortices. This increase in fast activity could either persist during 10–20 s, and be accompanied by an increase in delta and/or theta patterns (Borjigin et al., 2013), or be replaced by an enhancement of delta activity (1–5 Hz) lasting ~10 s (Schramm et al., 2020; Figures 2C1, 3C2). In these two models, the isoelectric phase (<10 mV, or decrease of at least 95% of the normoxic amplitude) was reached about 30–60 s after brain anoxia onset (Figures 2C1, 3C2; Borjigin et al., 2013; Lee et al., 2017; Schramm et al., 2020).

Insights into the neuronal substrate of anoxia-induced fast EEG activity were recently gained from the rat asphyxia model. By the means of *in vivo* intracellular recordings from neocortical pyramidal neurons, Schramm et al. (2020) found that the near-death high-frequency cortical oscillations correlated with repetitive membrane depolarizations (Figure 2C2, top) eliciting rhythmic cell firing (Schramm et al., 2020). Strikingly, these periodic depolarizations were individually composed of small amplitude high-frequency (from 100 to 140 Hz) membrane fluctuations having the shape of synaptic potentials, which were tightly correlated with the fastest oscillations in the EEG (Figure 2C2, top).

### 2.2. Fast activity: basic mechanisms

The cellular mechanisms subtending the anoxia-induced high-frequency synaptic activity in cortical cells remained not fully understood. However, several *in vitro* studies have pinpointed complex synaptic processes involving the modulation of both excitatory and inhibitory synapses. A progressive increase in the frequency (up to 50 Hz) of inhibitory currents, resulting from a Ca^2+^-dependent vesicular release of GABA, has been found in hippocampal pyramidal cells undergoing simulated ischemia *in vitro* (Allen and Attwell, 2004). Moreover, a short episode of gamma oscillations (20–40 Hz) has been observed at the beginning of the anoxic depolarization (see below) in CA3 pyramidal neurons as well as in the simultaneously recorded local field potential (Dzhala et al., 2001). These hippocampal high-frequency oscillations originated from adenosine- and potassium-dependent modulation of glutamate and GABAergic synaptic activities, synchronized through inhibitory synaptic processes mediated by GABA_A_ receptors (Dzhala et al., 2001).

Similar mixed synaptic events have been found *in vitro* in neocortical layer 5 pyramidal neurons after hypoxia (Fleidervish et al., 2001; Revah et al., 2016). Specifically, a barrage of high-frequency (up to 300 Hz) spontaneous excitatory and inhibitory post-synaptic currents was observed in somatosensory cortex pyramidal cells within the first minute of hypoxia (Fleidervish et al., 2001; Revah et al., 2016). This changes in synaptic activities are reminiscent of the asphyxia-induced high-frequency synaptic potentials found *in vivo* in the same cell type (Schramm et al., 2020; Figure 2C2 vs. Figure 2D).

The neocortical changes in fast activity are not sensitive to tetrodotoxin (a blocker of the voltage-gated sodium channels eliciting action potentials) suggesting that anoxia exerts a direct influence on the vesicular release mechanisms (Revah et al., 2016). Indeed, an early increase in transmitter release is associated with a large increase in intracellular Ca^2+^ concentration just after exposure to hypoxia (Revah et al., 2016). This increase in Ca^2+^ is likely triggered by the activation of glutamatergic receptors, probably resulting from an increased extracellular glutamate concentration after oxygen deprivation (Katchman and Hershkowitz, 1993; Fleidervish et al., 2001; Allen and Attwell, 2004). In agreement with the *in vitro* results, the mean membrane potential of neurons during the burst of rapid synaptic events *in vivo* was similar to that under pre-anoxic conditions, indicating that the ATPase activity governing homeostatic control of the resting membrane potential was not abrogated upon the increase in synaptic activity. Taken together, these results suggest that an early increase in extracellular glutamate is the essential trigger for the complex sequence of presynaptic and postsynaptic events that follows the onset of hypoxia and is responsible for high-frequency oscillations in cortical networks.

The transient surge of gamma oscillations in the EEG in response to brain anoxia is apparently paradoxical since this cortical pattern is especially energy demanding. Indeed, the amount of oxygen consumption during physiological gamma oscillations (30–90 Hz) in mice and rats is particularly elevated, approaching that associated with episodes of epileptic activity (Kann et al., 2011). Thus, it is plausible that the burst of high-frequency activity during the dying process is not merely due to the breakdown of cell metabolism but also results from additional brain mechanisms that remain to be elucidated.

### 2.3. Slow oscillations

The first account of hypoxia-induced slow cortical oscillations was given by Hans Berger, the discoverer of the human EEG. He found that inhalation and exhalation of a sealed bag through a trap that absorbed exhaled CO_2_ caused a rapid loss of consciousness, which was correlated with fairly abrupt changes in cortical waveforms, from the alpha pattern to high-amplitude slow waves (Berger, 1934). Berger, who was deeply impressed by its discovery, vowed never to repeat the experiment. Consistent with this pioneering study, numerous clinical and experimental investigations have shown that the anoxia/ischemia-induced fast cortical pattern is generally followed by slow oscillations in the delta frequency (1–5 Hz) range. This has been consistently demonstrated in birds (Raj et al., 1991), mice (Wang et al., 2015), rats (Borjigin et al., 2013; Lee et al., 2017; Schramm et al., 2020; Figure 2C, bottom panels), and cats (Sugar and Gerard, 1938; Hossmann and Kleihues, 1973), with a delay from the onset of the anoxic insult ranging from 10 to 60 s. Slow cortical patterns have been also found in humans during brief cardiac arrest (Clute and Levy, 1990) and vasovagal syncope accompanied by asystole or extremely low blood pressure (Ammirati et al., 1998; Figure 3A).

The detailed mechanisms by which oxygen deprivation produces low-frequency oscillations in the neocortex remain unclear. However, *in vitro* studies and computer modeling highlighted a key role of intrinsic ionic conductance such as the K^+^_ATP_ channels, activated by a decrease in the intracellular concentration of ATP. It has been shown from entorhinal cortical slices that modulation of K^+^_ATP_ channels governed low-frequency oscillations at both single-cell and network levels and that tolbutamide, a blocker of these channels, suppressed slow rhythmic activity (Cunningham et al., 2006). Moreover, a more recent network model of leaky integrate-and-fire neurons with an additional dependency on cell metabolism (Joo et al., 2021) showed that when the rate of ATP production declines, the neural network is engaged in synchronized oscillatory behavior, eventually entering the delta range, as observed after oxygen deprivation *in vivo*. It can thus be hypothesized that subtle interactions between ATP-sensitive K^+^ channels and excitatory synapses initiate slow fluctuations in the membrane potential of neurons and the synaptic networks during metabolic failure.

## 3. The near-death isoelectric state

### 3.1. Phenomenology

If the lack of brain oxygenation persists, the slow cortical waves, which gradually decay in amplitude and frequency, are the prelude to an isoelectric EEG, i.e., a cortical state devoid of any spontaneous activity. The maximum amplitude of an isoelectric EEG, usually in the tens of microvolts range, is variable according to the authors, and depends on the gain of the recording systems. It is therefore an equivocal criterion. A valid assessment of an isoelectric EEG must be the absence of underlying electrophysiological events in subjacent neurons, demonstrating genuine electrocerebral inactivity (Altwegg-Boussac et al., 2014, 2016).

In the asphyxia rat model, the period of isoelectric EEG immediately following the slow oscillations was accompanied by a drastic reduction in heart rate, undetectable arterial oxygen saturation (SpO_2_), and pupil dilation (Schramm et al., 2020). Similar correlations were found in patients in whom electrocerebral inactivity, caused by heart failure or carotid endarterectomy, was related to profound hemodynamic and metabolic defects. In the pioneering studies examining EEG changes accompanying ventricular fibrillation (Clute and Levy, 1990), the last systolic blood pressure was found to be concomitant with rapid attenuation of cortical signals and loss of delta activity, ending in an isoelectric EEG in the absence of active defibrillation. Cardiac arrest during a vasovagal reflex rapidly causes a flat EEG that correlates with a collapse of arterial pressure (Ammirati et al., 1998; Figure 3A); and the lowest regional cerebral oxygen saturation (rSO_2_ < 15%) measured in patients undergoing cardiopulmonary resuscitation was reliably associated with voltage suppression in their EEG (Reagan et al., 2018). Finally, a recent study (Matory et al., 2021), in which EEG, cerebral blood flow (CBF), and electrocardiogram were monitored simultaneously in patients at the time of cardiac arrest, indicated that electrocerebral inactivity occurred in most cases before or in conjunction with the last heartbeat, but after CBF was stopped (Figure 3B).

These findings indicated a close relation between EEG changes and CBF (Sharbrough et al., 1973; Foreman and Claassen, 2012). The isoelectric state is associated with a decrease in CBF toward the infarction threshold (10–12 mL/100 g/min and below), and therefore a dramatic impairment of cerebral oxygen metabolism (Nagata et al., 1989). These congruent animal and human data suggest that the dissolution of spontaneous cortical activities upon cardiorespiratory failure, causing inactive EEG, is directly due to the fall of brain oxygenation.

### 3.2. Mechanisms and functional consequences

An isoelectric state can occur after many other brain injuries, such as severe head trauma (Husain, 2006), profound hypothermia (Stecker et al., 2001), or administration of elevated doses of CNS depressant medications, such as barbiturate-derived drugs (Altwegg-Boussac et al., 2014, 2016, 2017; Figure 3C1) and halogenated volatile anesthetics (Kroeger and Amzica, 2007; Kroeger et al., 2013; Carton-Leclercq et al., 2021). Regardless of its etiology, this electrocerebral inactivity reflects a complete cessation of spontaneous neuronal activity, at least in the cerebral cortex, including action potentials and synaptic events. This has been directly demonstrated by simultaneous EEG and intracellular recordings, which consistently show an absence of spontaneous fluctuations (Kroeger and Amzica, 2007; Kroeger et al., 2013; Altwegg-Boussac et al., 2014, 2016, 2017). When induced by drugs, the isoelectric state is attained after a relatively long delay (5–35 min) following the bolus injection (Altwegg-Boussac et al., 2017; Carton-Leclercq et al., 2021). It is the result of a neuronal over-inhibition, involving synaptic and ion channel mechanisms, which progressively suppress ongoing activities in the neuronal networks, without canceling cell excitability or precluding sensory-evoked post-synaptic responses (Figure 3C1; Altwegg-Boussac et al., 2014, 2017; Carton-Leclercq et al., 2021).

In the case of oxygen deprivation, the timing and mechanisms of electrocerebral silence are fundamentally different. In the rat *in vivo*, asphyxia (Schramm et al., 2020) or global brain ischemia (Xu and Pulsinelli, 1994) cause a much faster (within the first 40 s) vanishing of spontaneous synaptic and firing activities in cortical neurons, which parallels the dissolution of EEG waveforms (Figure 3C2; Schramm et al., 2020). This is in accordance with the fast suspension of spontaneous excitatory and inhibitory synaptic currents found *in vitro* in hippocampal (Zhu and Krnjević, 1994) and neocortical pyramidal cells after anoxia (Rosen and Morris, 1993; Peña and Ramirez, 2005). The anoxia-induced fading of background synaptic activity can result from multiple presynaptic defects, including a reduction of presynaptic Ca^2+^ currents and/or of impulse conduction block (Young and Somjen, 1992), a decrease in synapsin-I phosphorylation (phosphorylation dissociates vesicles from actin) causing a decline in neurotransmitter release and an augmented release of adenosine which, in acting on A1 receptors, induces a depression of synaptic transmission (Arlinghaus and Lee, 1996; Gervitz et al., 2001). Although the consecutive reduction of postsynaptic conductance is expected to considerably increase the global cell resistance (Destexhe et al., 2003), the early period of the anoxia-generated isoelectric state is accompanied by a decrease in membrane resistance (Figure 3C2, bottom; Xu and Pulsinelli, 1994; Schramm et al., 2020). This may result from an additional membrane conductance caused by the opening of ATP-dependent K^+^ channels, which are rapidly activated by falling ATP and known to reduce neuronal resistance upon oxygen deprivation (Jiang and Haddad, 1997; Fleidervish et al., 2001). This increase in K^+^ currents probably also participates in the fall of synaptic drive and the net cellular hyperpolarization observed in cortical neurons during the establishment of the isoelectric state (Fujimura et al., 1997).

## 4. The wave of death

### 4.1. Basic features

**Figure 4:**
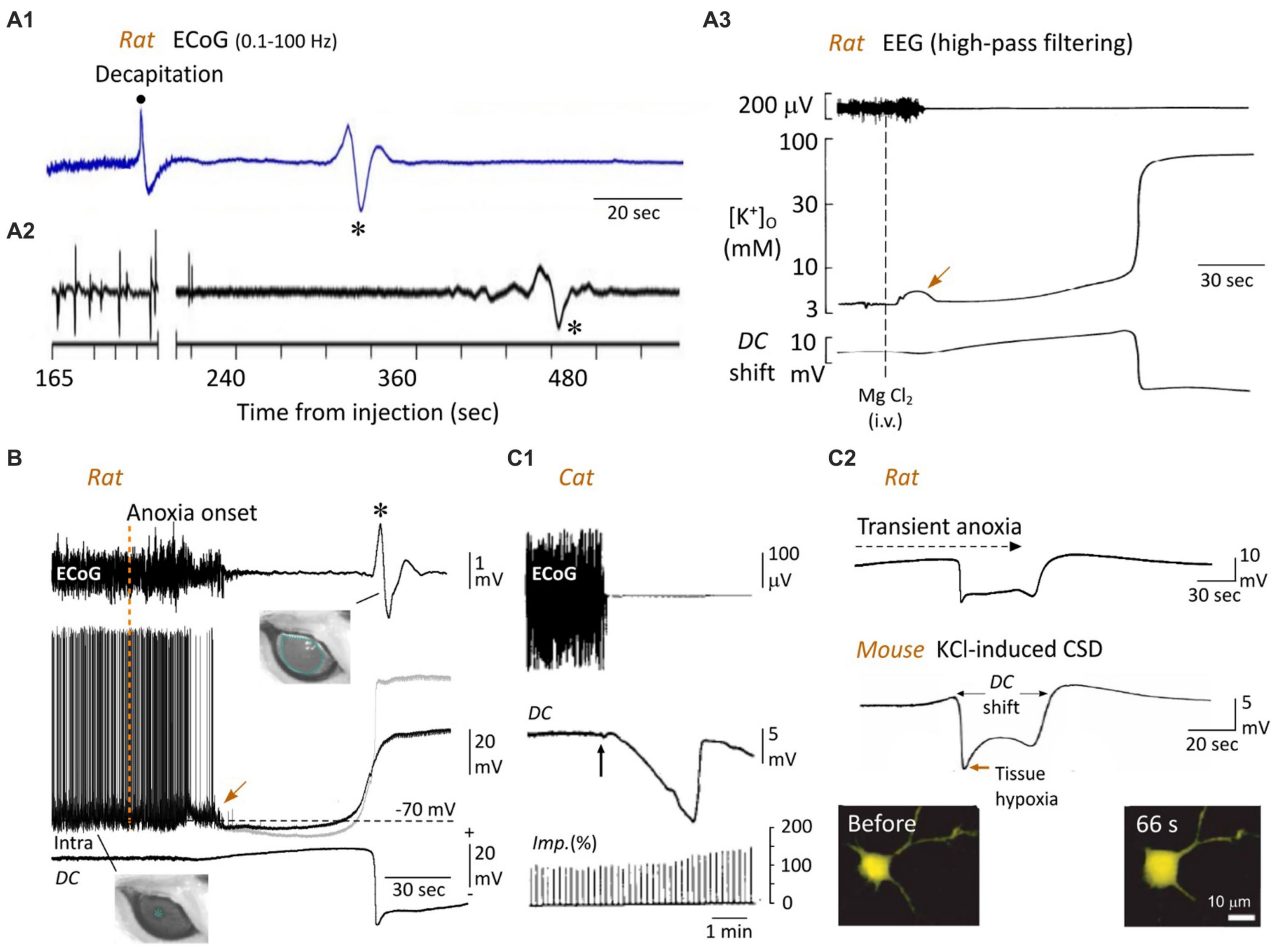
The WoD and the neuronal anoxic depolarization. **(A)** Cortical WoD and its attendant intracortical *DC* shift. ECoG recordings from an awaked rat before and after decapitation **(A1)** and from another one euthanatized with an overdose of pentobarbital **(A2)** (the time after injection of 120 mg/kg is indicated). Note the WoDs (asterisks) occurring on a flat EEG with various time delays. In **(A2)**, the EEG waveforms before the break of the abscissa represent a typical burst-suppression pattern. **(A3)** EEG activity, interstitial electrical potential (*DC* potential) of rat brain neocortex, and arterial blood pressure after cardiac arrest (arrow), which was induced by intravenous injection of saturated solution of MgCl. Here, the WoD is not detected due to inadequate filtering of the EEG record. The corresponding concentration of interstitial K^+^ (measured by K^+^-sensitive microelectrode) is depicted. The negative deflection in the *DC* potential tightly correlates with the sudden rise in extracellular K^+^ (**A1,A2** are modified from Van Rijn et al., 2011; **A3** from Hansen, 1978). **(B)** Simultaneous recordings of ECoG (top), intracellular activity from a subjacent pyramidal neuron (middle) and local *DC* potential (bottom) in the rat, from normoxic period and during oxygen deprivation produced by asphyxiation (vertical dashed line). The gray trace is the Vm corrected with the corresponding extracellular *DC* potential. Note the near-simultaneous occurrence of surface WoD (asterisk), *DC* shift and neuronal anoxic depolarization. A large, non-reactive, mydriasis correlates with the WoD, suggesting a loss of brainstem functions at the time of the cortical anoxic depolarization (modified from Schramm et al., 2020). Note the jump in membrane potential (arrow), which coincides with the early and transient increase in extracellular K^+^ (**A3**, arrow). **(C)** Bain anoxia causes neuronal swelling. **(C1)** Relationship between ECoG activity, cortical steady potential (*DC*) and intracortical impedance (Imp) in the cat, before and during ischemia after clamping brain arteries (vertical arrow). Such an increase in cortical resistivity, correlating with the *DC* potential, is due to shrinkage of extracellular space. **(C2)** In the mouse cortex *in vitro*, topical application of a high concentration of K^+^, which causes a *DC* shift similar to that induced by anoxia (*top*, modified from Schramm et al., 2020), is accompanied by a marked increase in cell body volume of neurons, as shown at the indicated time before the onset of *DC* potential (modified from Takano et al., 2007). In **(A1)**, the band pass of ECoG records is indicated (same as in **B**, top record).

When a critical level of metabolic failure is reached after oxygen deprivation, corresponding to 13–18% of the normal level of ATP (Verhaegen et al., 1995), the flatline EEG is transiently disrupted by a slowly-developing polyphasic wave, displaying a positive–negative–positive shape due to classical high-pass filtering (0.1 Hz) of EEG (van Rijn et al., 2011; Schramm et al., 2020; Figures 4A,B). The delay of the occurrence of this near-death cortical potential, termed “wave of death” (WoD) in rodents (van Rijn et al., 2011; Schramm et al., 2020) or “terminal spreading depolarization” (TSD) in humans (Carlson et al., 2018; Dreier et al., 2018), varies between species and depends on the physiological/experimental conditions and the origin of the loss of brain oxygenation. This phenomenon differs from the cortical spreading depression, as it is due to metabolic impairment of brain tissue, with anoxic depolarizations (see below) that are systematically preceded by a silencing of ongoing cortical activities and may cause cell death (Pietrobon and Moskowitz, 2014).

In patients with devastating brain injury, engaged in a Do Not Resuscitate–Comfort Care protocol, the terminal extubation, which caused a slow collapse of arterial pressure and tissue partial pressure of O_2_, provoked a TSD in the ECoG 13–266 s after the silencing of cortical electrical activity (Dreier et al., 2018). *In vivo* in rodents, the WoD is expected to occur between 50 and 120 s after oxygen deprivation. The shortest delay (50 s) is observed after global brain ischemia provoked by decapitation in the awake rat (Figure 4A1). This latency is significantly augmented when decapitation is performed on previously anesthetized animals (~80 s) (van Rijn et al., 2011) and further increased when death is prompted by a lethal dose of anesthetics (~90 s) rather than decapitation (Figure 4A2; van Rijn et al., 2011), probably because of the time taken by depressant drugs to block the brainstem respiratory centers. WoD latency is also delayed by hypothermia (Verhaegen et al., 1995; Jarvis et al., 2001), most likely due to the reduced metabolic demand. In the rodent asphyxia models, the WoD appeared 50–120 s after interruption of artificial ventilation (Figure 4B, top record), without significant difference between the different cortical areas, and coincides with the minimal level of SpO_2_ (Takanashi et al., 1991; Schramm et al., 2020). The WoD has never been observed in cell cultures and occurs after 1–12 min of metabolic failure in cortical slices, depending on the glucose level in the incubation medium (Lipton, 1999).

Although the WoD (or TSD) appears to be a limited event in time (due to *ad hoc* filtering), it reflects the onset of a sudden and prolonged large-amplitude (from −5 to −30 mV) negative deviation of the intracerebral direct current (*DC*), classically called *DC* shift (Hansen, 1985; Lipton, 1999; Somjen, 2004). While Leão initially described neocortical *DC* shift in response to focal ischemia (Leao, 1947), Bures and Buresova (1957) were the first to directly demonstrate its occurrence in the cortex of animals dying from anoxia or ischemia. They named it “anoxische terminal depolarization,” assuming (erroneously) that such a very large negative voltage shift was the hallmark of irreversible brain death. Usually preceded by a slowly developing positive potential, neocortical *DC* shift is characterized by an early negative peak, followed by a less negative plateau and a second negative peak, which finalizes the voltage drop (Hansen, 1978; Schramm et al., 2020; Figures 4A3,B,C2, 5). The relationship between the WoD and the *DC* shift was directly demonstrated by simultaneous EEG and intracortical local field potential recordings in the rodent asphyxia model (Figure 4B; Schramm et al., 2020), demonstrating that the normal compartmentalization of ions between the intra- and extra-cellular spaces breaks down at the occurrence of the WoD in the cerebral cortex.

**Figure 5:**
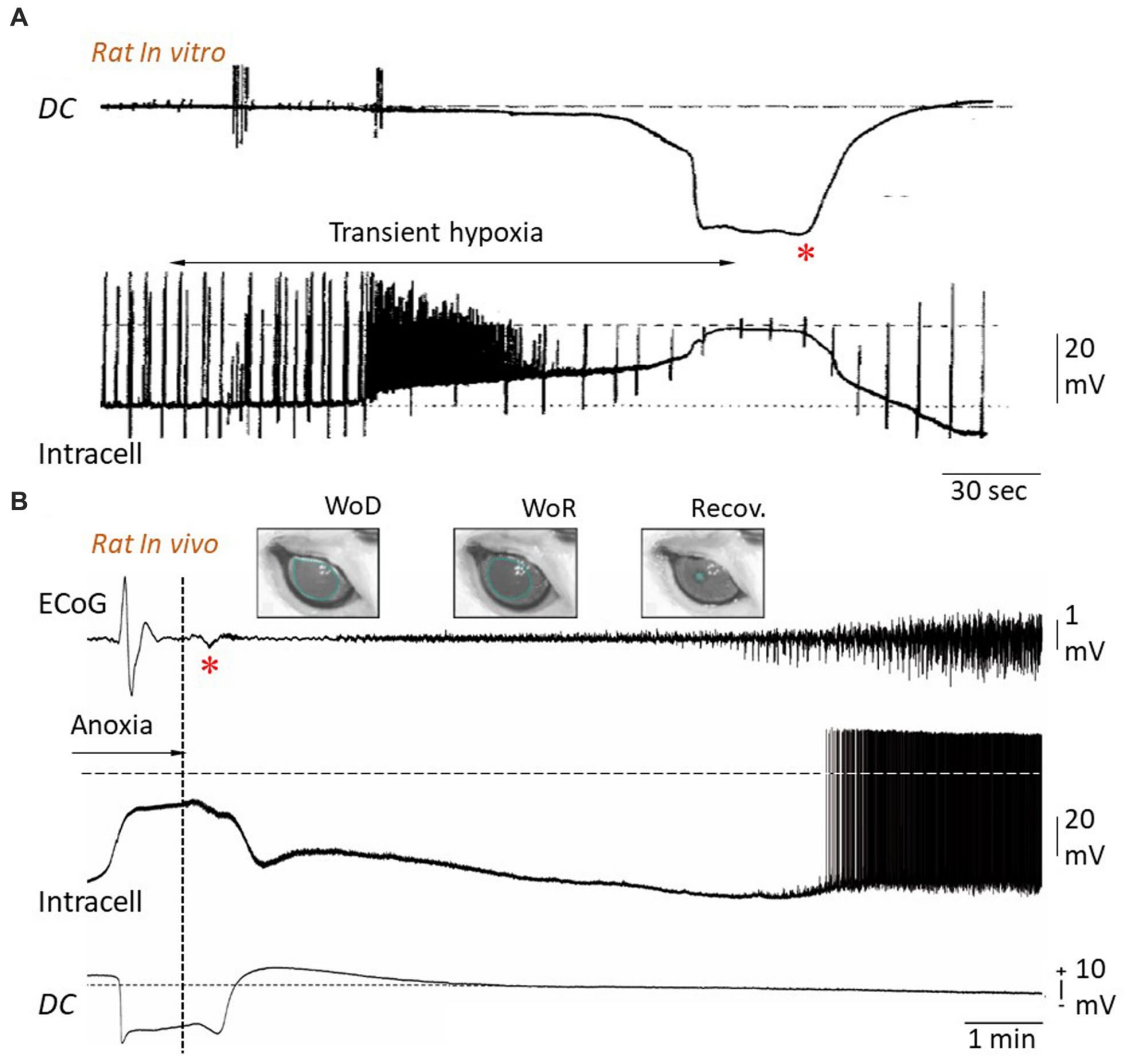
Reversibility of anoxic depolarization. **(A)** Simultaneous extra- and intracellular potential changes in rat hippocampal slice during transient hypoxia. Hypoxia causes a negative *DC* shift in the extracellular medium (upper trace) together with a neuronal depolarization, eliciting a burst of action potentials, until a cellular AD is established (lower trace). Restoration of oxygen supply results, after about 20 s, in a progressive cancelation of extracellular negativity concomitantly with repolarization of the neuronal membrane. The up and down deviations in the intracellular recording are caused by injected current pulses used to test membrane impedance and excitability. The horizontal dashed line indicates zero potential (modified from Czéh et al., 1993). **(B)** Simultaneous recordings of ECoG (top), intracellular activity from a subjacent pyramidal neuron (middle) and intracortical *DC* potential (bottom) in the rat, during oxygen deprivation produced by asphyxiation and after resumption of oxygen supply (vertical dotted line). The cancelation of *DC* negativity, which occurs conjointly with restoration of neuronal membrane potential, is correlated with a small polyphasic wave in the corresponding ECoG (asterisk). Since this wave systematically correlates with a subsequent recovery of neuronal and ECoG patterns, it was called “Wave of Resuscitation” (WoR). WoR is also correlated with a progressive retrieval of pre-anoxic pupil diameter (see Figure 4B), which fully recovers ~20 min after resuscitation onset (Recov.) (modified from Schramm et al., 2020).

### 4.2. Mechanisms

WoD and *DC* shift are both concomitant with a near-complete membrane depolarization of cortical neurons (Figure 4B, middle record; Lipton, 1999; Pietrobon and Moskowitz, 2014; Schramm et al., 2020), called anoxic depolarization (AD), which is caused by an abrupt, nonlinear, dissolution of ion gradients across the cell membrane. Elegant theoretical studies by Zandt et al. (2011) and van Putten (2020) showed that the peculiar WoD pattern, recorded in decapitated (van Rijn et al., 2011) and asphyxiated (Schramm et al., 2020) rats, could be predicted from Hodgkin-Huxley modeling of neuronal membrane currents after the cessation of Na^+^-K^+^ pump activity. In particular, they found that the simulated sudden depolarization of cortical neurons, as observed during AD, combined with a high-pass filter of the EEG, led to a cortical wave similar to WoD. This biophysical model is consistent with simultaneous recordings of WoD and the intracellular activity of underlying cortical neurons (Schramm et al., 2020), as well as with the well-known basic mechanisms of cellular AD.

It is well established that AD is primarily due to a fall in ATP concentration induced by oxygen deprivation, which rapidly impairs the activity of the Na^+^-K^+^-ATPase involved in the maintenance of the resting membrane potential and transmembrane gradients for Na^+^ and K^+^ in neurons (Lipton, 1999; Pietrobon and Moskowitz, 2014). This hypothesis is strongly supported by the fact that the application of low concentrations of Na^+^-K^+^ ATPase antagonist (ouabain), to mimic the levels of ATPase pump inhibition obtained during anoxia, induced large neuronal depolarization resembling AD (Balestrino, 1995; Lipton, 1999). As mentioned above, the early decrease in ATP is also responsible for the opening of ATP-dependent K^+^ channels (Fleidervish et al., 2001), resulting in (along with the activation of Ca^2+^-dependent K^+^ channels, see Liu et al., 1999) a net neuronal hyperpolarization preceding the AD in neocortical neurons (Figure 4B, middle, gray trace).

Dysfunction in Na^+^-K^+^-ATPase activity largely compromises the homeostatic control of cell resting membrane potential. This causes a net efflux of K^+^ and an increase in its extracellular concentration (Figure 4A3; Hansen, 1978, 1985), probably supplemented by an increase in K^+^ conductances (Martin et al., 1994). The early rise in extracellular K^+^ (of about 3–10 mM) (Hansen, 1985; Martin et al., 1994; Pietrobon and Moskowitz, 2014; Figure 4A3, middle trace), due to the high dependence of cell resting membrane potential on the K^+^ gradient, provides an electrochemical force that facilitates membrane depolarization. It is also likely that the activation of K^+^ channels involved in action potential repolarization will contribute to additional K^+^ efflux and further depolarization when the membrane potential reaches the firing threshold (Zandt et al., 2013). From this point on, voltage-dependent Na^+^ and Ca^2+^ channels, as well as glutamatergic receptors, will be synergistically involved in the generation of a self-sustaining inward current and, consequently, in the full development of the AD (Lipton, 1999; Pietrobon and Moskowitz, 2014).

The cooperative role of cationic currents was evidenced by measurements of a concomitant increase in K^+^ and a decrease in Ca^2+^ and Na^+^ in the interstitial medium, which correlated with the rapid negative *DC* shift during anoxia (Hansen, 1985; Xie et al., 1995; Lipton, 1999; Pietrobon and Moskowitz, 2014). The crucial implication of Na^+^ currents was demonstrated by the blockade of AD by various voltage-dependent Na^+^ channels blockers, such as TTX, lidocaine, or dibucaine (Aitken et al., 1991; Weber and Taylor, 1994; Douglas et al., 2011) and its delayed occurrence by a reduction in extracellular Na^+^ (Müller and Somjen, 2000). AD is also delayed under conditions of low extracellular Ca^2+^ (Tanaka et al., 1997), and significantly impeded by the inhibition of voltage-dependent Ca^2+^ channels (Jing et al., 1993). In addition, a complete blockade of AD in hippocampal slices can be achieved by a combination of cationic channel inhibitors and NMDA and AMPA receptor antagonists (Müller and Somjen, 2000; Pietrobon and Moskowitz, 2014). Since the Na^+^-K^+^-ATPase compensates for leak currents, it is expected that its functional failure would no longer be able to counteract passive ion flows across the neuronal membrane. This should ultimately lead to a loss of concentration gradients of all diffusible ions, bringing the membrane potential to zero. However, the membrane potential reached during the AD plateau remains slightly negative. This is because the inside of the cell contains many negatively charged proteins that cannot pass through the membrane, causing a ubiquitous phenomenon in living cells known as the Gibbs-Donnan effect (Overbeek, 1956). Theoretical calculation of the Gibbs-Donnan potential at the AD plateau, assuming electroneutrality on both sides of the cell membrane and an intracellular concentration of impermeant and negatively charged macromolecules of 148 mM (Dijkstra et al., 2016), results in a steady membrane potential of ~ −20 mV, a value that closely matches that measured *in vivo* in cortical cells (Figure 4B, gray trace; Xu and Pulsinelli, 1994; Schramm et al., 2020).

In addition to electrical changes seen at various scales in the brain, including single cell (AD) and local field potential (*DC shift*), the WoD also correlates with a set of biochemical, thermic, structural and functional modifications that characterized the dying brain.

### 4.3. WoD-related biochemical and thermic changes

A decrease in intracellular pH has been found to precede AD both *in vivo* (Silver and Erecińska, 1990) and *in vitro* (Martin et al., 1994), even though ATP levels are still relatively high. This change in intracellular pH to oxygen deprivation is independent of changes in intracellular Ca^2+^ and may instead reflect an alteration in the activity of the acid-extruding Na^+^/H^+^ exchanger and overactivation of a proton conductance during AD (Diarra et al., 1999).

Changes in nerve tissue temperature during anoxia-induced depolarization have been originally described by Tasaki and Byrne (1991) in the isolated retinae of amphibians using highly sensitive heat sensors. They found that a rise in temperature, from 5 to 15 mdeg/min, invariably preceded the onset of the *DC* shift at the recording site and traveled along the retinae at ~4 mm/min, namely at the speed at which AD was known to travel. In patients with intracerebral hemorrhage, a small increase in brain temperature by 0.2°C (not core temperature) is observed before the anoxic waves and a return to the baseline value is reached after 35 min (Schiefecker et al., 2018). Although the origin of anoxia-induced elevation of brain temperature remains unclear, theoretical models suggest that heat production is due to loss of free energy (Dreier et al., 2013): the Gibbs free energy, a thermodynamic potential that is displaced when a system reaches chemical equilibrium, such as after the loss of ATP-dependent ion pump function during AD.

### 4.4. WoD-associated structural modifications

The pathological Gibbs–Donnan equilibrium that sets the steady membrane potential during AD is also responsible for alterations in the morphology and size of the neurons and the electrical resistivity of the brain tissue. The metabolic impairment induced by anoxia, which results in depletion of intracellular ATP and cessation of active ion transport, leads to a dissipative redistribution of diffusible ions across the neuronal membrane, but in the presence of non-permeable intracellular polyanions. This provokes an increase in osmotic pressure inside the cell relative to the interstitial one. Water will therefore enter the cell, through aquaporins, and the cell will swell. It is expected that a massive swelling of neurons will cause shrinkage of interstitial space, leading to an increase in the electrical resistivity of anoxic brain tissue. This phenomenon was indirectly, and astutely, first described by Freygang and Landau (1955) who found that transcortical resistivity, as measured by the potential change produced across the cortex by a constant current pulse, was increased during the cortical wave generated by an artificial increase in extracellular K^+^. In this pioneering study, the maximal rise in cortical resistivity coincided with the peak negativity of the *DC* shift, i.e., when neurons have reached the AD plateau. Similar correlations were obtained in rabbits after brain circulation arrest (Ochs and Van Harreveld, 1956) and in cats following the clamping of subclavian arteries or lowering (<100 mm Hg) of the systemic blood pressure (Hossmann, 1971). In the latter study, the impedance of the cortical tissue was increased by 20–30% at the peak of the *DC* shift (Figure 4C1), while the cortical responses to pyramidal tract stimulation were still present suggesting the persistence of axonal propagation of action potentials. The original assessment of change in the extracellular space in relation to the increase in brain resistivity was based on the Maxwell approach (Fenstermacher et al., 1970), using the following equation:


r/2=(1−R/R1)/(2+R/R1),

where r is the volume fraction of the cortex occupied by cells; R1 is the specific resistance of the cortex, and R is the specific resistance of the intercellular fluid. According to this biophysical model, the extracellular space was reduced from 21 to 15% (Hossmann, 1971). Such a shrinkage of the extracellular compartment was shown to reflect an equivalent cellular swelling due to water displacement into the intracellular compartment to maintain the osmotic balance between the two media (see above). The enlargement of cell volume is probably worsened by the intracellular production of osmotically active molecules, such as lactic acid, by anaerobic metabolism (Tranum-Jensen et al., 1981). Other quantifications, based on changes in ionic diffusion parameters, indicated that the volume fraction of the extracellular space fell to 5% during terminal spinal cord anoxia (Syková et al., 1994; Syková and Nicholson, 2008).

Imaging techniques have also been applied to the mapping of anoxia-induced depolarization by measuring associated changes in intrinsic optical signals due to cellular swelling. These measurements of light transmittance changes in the cortical tissue submitted to oxygen deprivation also concluded that *DC* shift correlated with a shrinkage of interstitial space (Turner et al., 1995; Obeidat and Andrew, 1998; Kreisman et al., 2000). Finally, the direct relationship between extracellular space shrinkage and structural changes in neurons was established by measuring the cross-sectional area of layer 2 cortical neurons during K^+^- or anoxia-induced *DC* shift, which indicated a 37% increase in cell body volume (Takano et al., 2007; Figure 4C2). This was accompanied by morphological changes to the dendrites in the seconds after AD, ranging from extensive blebbing to loss of spines (Hasbani et al., 2001; Takano et al., 2007), which could be reversed upon restoration of sufficient oxygen and glucose levels (Hasbani et al., 2001; Somjen, 2004; Takano et al., 2007). In the most extreme conditions, the neuronal membrane may become incontinent, meaning that its contents, including all normally impermeable components, escape in the interstitial tissue (lysis), an irreversible terminal state heralding cell death (Rothman, 1985).

### 4.5. Functional consequences

The loss of homeostatic regulation of transmembrane ion concentrations and the associated increase in Na^+^, K^+^, Ca^2+^, and Cl^−^ conductances during WoD and TSD are expected to impact the neuronal integrative properties, including membrane resistance, which controls the voltage response to input current, and membrane time constant, which represents the efficiency with which the neuron sums synaptic events over time. It has thus been demonstrated *in vitro* from oxygen-deprived hippocampal and neocortical neurons (Hansen et al., 1982; Snow et al., 1983; Czéh et al., 1993; Brisson and Andrew, 2012; Figure 5A) as well as *in vivo* from neocortical pyramidal cells following asphyxia (Schramm et al., 2020; Figure 5B), that membrane input resistance and time constant are nearly abolished during AD. Interestingly, the time course of the fall in membrane resistance parallels the development of AD (Czéh et al., 1993; Brisson and Andrew, 2012; Schramm et al., 2020; Figure 5A), reflecting thus the progressive increase in ionic conductance induced by the metabolic impairment. Accordingly, the collective collapse of membrane resistance and time constant culminates at the plateau of AD, when the membrane potential is set by the pathological Gibbs-Donnan equilibrium in the absence of ATPase activity. The loss of integrative properties during AD is accompanied by a progressive reduction in action potential amplitude (Czéh et al., 1993; Brisson and Andrew, 2012; Zandt et al., 2013; Figure 5A), likely due to the inactivation of Na^+^ channels over the anoxic depolarization, until reaching a complete cessation of cell firing at the plateau potential, marking a loss of neuronal excitability.

WoD, along with the underlying *DC* shift and cellular AD, thus provide multi-scale electrophysiological markers of drastic alterations in brain functions, since they consistently correlate with (1) an isoelectric EEG, i.e., when the thalamocortical system no longer generates spontaneous activity, (2) a complete absence of synaptic activity and ongoing firing in injured neurons, (3) a collapse of neuronal integrative properties and intrinsic excitability, rendering neurons unable to process external information, (4) a dramatic drop in intracellular and extracellular pH, (5) a slight increase in brain temperature and, (6) a change in cell morphology, including soma swelling and dendritic alterations, leading to an increase in brain electrical resistivity. The functional impact of increased brain resistance is not clear. However, it could artificially amplify the amplitude, and distort the shape, of the WoD and *DC* shift, because both potentials are generated by current flows through the shrunken interstitial space of the brain.

Collectively, these pathological cerebral events are expected to cause a complete dissolution of all brain processes, as ongoing brain activity is interrupted, and neural networks are unable to integrate and process information. However, the various brain functions will not collapse simultaneously. It was shown from *in vitro* investigations that activity of Na^+^-K^+^-ATPase of higher-brain neurons (cortical, thalamic, and striatal) fails promptly compared to hypothalamic and brainstem neurons, which are naturally more resistant to anoxia (Brisson and Andrew, 2012; Brisson et al., 2013, 2014). As a result, functional impairment of different neuronal populations should occur *in vivo* with variable delays after brain anoxia, depending on the structure involved, with a sequential pattern of AD from higher to lower brain regions. However, recent findings from an asphyxia rodent model showed that the dilatation of the animal’s pupil reaches maximal a-reactive mydriasis at the time of the cortical WoD, suggesting a disruption of the third cranial nerve nucleus function (Ritter et al., 1999) concomitant with the dissolution of the neocortical functions. To elucidate the genuine spatiotemporal sequence of AD in the brain, it will be necessary to perform simultaneously *in vivo* multisite brain recordings during systemic anoxia.

## 5. Reversibility of anoxic depolarization and the wave of resuscitation

The WoD, through its attendant cellular defects including decreased ATP levels and increased intracellular concentration of free fatty acids, Na^+^, and Ca^2+^, is a readily recognizable predictor of an acute or delayed death of neurons (Lipton, 1999; Somjen, 2004). However, despite a common belief (Toyoda et al., 2021), WoD and cellular AD are not inherently irreversible, and the duration of the anoxic period after which oxygen replenishment no longer allows for neuronal recovery is both cell type-dependent and variable within each cell population (Somjen, 2004; Brisson and Andrew, 2012; Juzekaeva et al., 2020). Recovery from AD has been demonstrated by Czéh et al. (1993) from hippocampal slices subjected to periods of hypoxia (5% N2/5% CO_2_) lasting no longer than 5 min. These authors showed that restoration of a normoxic condition during AD caused, within ~30 s, the cancelation of the local negative *DC* potential and the repolarization of the neuronal membrane, which was accompanied by a recovery of membrane resistance and cellular excitability (Figure 5A). More recently, it was demonstrated *in vitro* that nearly half of layer 4 neocortical neurons survived an anoxic episode of less than 10 min and that the probability of finding live neurons decreased by about 2% for each additional minute of oxygen–glucose deficiency (Juzekaeva et al., 2020). Neurons located in supragranular layers were shown to exhibit nearly threefold higher resistance to anoxia than layer 4 neurons. These findings demonstrate that: (1) the neuronal AD in the cortex *in vitro* is reversible, (2) the time to reach the point of no return (irreversible neuronal damage) depends on the brain structure, (3) the rate of cell survival depends on the duration of the anoxic episode and, (4) different cell types within the same structure have a differential vulnerability to anoxia.

Reversibility of the neuronal AD has also been demonstrated *in vivo*. In the rodent asphyxia model, reinstatement of brain oxygenation 2–4 min after the onset of anoxia (i.e., in the course of AD) induced, in approximately 60% of cases, a slow recovery of the membrane potential of pyramidal neocortical neurons toward physiological resting potential values and the cancelation of the corresponding negative *DC* shift (Schramm et al., 2020; Figure 5B). This was likely due to the restoration of transmembrane ionic gradients, prompted by the renewal of ATP production and subsequent activation of Na^+^-K^+^-ATPase. The ionic flow generated in the interstitial medium during cell repolarization could be captured as a low-amplitude slow wave at the brain surface (Figure 5B, asterisk). This wave was thus termed the “wave of resuscitation” (WoR) (Schramm et al., 2020), as it systematically inaugurated: (1) the recovery of membrane polarization, excitability and integrative properties of neocortical neurons, (2) the regain of background synaptic activity, allowing spontaneous cell firing, (3) the return of ECoG activity and, (4) the recuperation of normal pupil diameter and pupilar reflex (Schramm et al., 2020; Figure 5B). The cortical WoR indicates not only the recovery of cortical activity but also the return, at least partially, of brainstem functions.

The WoR, therefore, provides an easily recordable cortical wave, highly predictive of the post-anoxic recovery of brain activities (without prejudging possible enduring brain damage) (Rossetti et al., 2016), which should be detectable in human patients during successful resuscitation procedures. An identification, or a lack of detection, of the WoR in patients, may thus contribute to the prognosis of a good post-anoxic outcome or the diagnosis of irreversible brain deterioration.

## 6. Contrasting effects on consciousness

Previous reports indicate complex, and seemingly contradictory, effects of oxygen deprivation on conscious processes. Indeed, a sustained brain anoxia is associated with a profound and possibly permanent state of unconsciousness which may be preceded in some cases by the perception of a vivid and lucid conscious experience (called a near-death experience or NDE). The neuronal mechanisms subtending anoxia-induced loss of consciousness and NDE are not yet fully explained.

### 6.1. Onset of terminal unconsciousness

As described above, the early surge of high-frequency patterns in EEG activity after oxygen deprivation is followed by a progressive suspension of cortical activities leading to an interruption of brain functions, including the level and content of consciousness (Luft and Noell, 1956). The timing and the characteristics of this loss of conscious processes remain unclear due to limited research on real-time correlations between behavior and brain activities in such a condition. Only one study has documented the instantaneous effect of cerebral circulation arrest on consciousness in humans. In an unethical study, Kabat (1943) induced transient brain ischemia in schizophrenic patients and inmates by compressing their common carotid and subclavian arteries, so that all circulation to the brain was cut off. This led to eyeball fixation and a narrowing of the visual field within 5–7 s after brain ischemia onset. A second later, the head fell on the chest and the subject slumped in his chair, attesting to the loss of consciousness. The corresponding EEG activity was dominated by slow waves and correlated with a loss of cornea1 reflexes (after ~10 s) (Kabat, 1943). These findings are consistent with other pioneering studies showing that when a normal subject breathes pure nitrogen (Gibbs et al., 1935) or is exposed to rapid decompression causing brain anoxia (Luft and Noell, 1956), it becomes rapidly unconscious or extremely confused, with a prevalence of low frequency (1–5 Hz) waves in its EEG.

The study of real-time modifications in conscious processes during life-threatening cardiac arrest is not feasible. However, experimentally induced syncope, which consists of a transient loss of consciousness (TLOC), provides an alternative means for exploring the relationship between cardiovascular failure, changes in cortical activity, and unconsciousness (Wieling et al., 2009). Vasovagal syncope, the most common form of reflex syncope, can be unmasked by the tilt table test which causes an increase in vagal tone instigating a cardiac pause of 3–52 s (Saal et al., 2017). Asystole either coincides (Ammirati et al., 1998; Figure 3A) or precedes the onset of TLOC (defined as either head-dropping, eye-opening, or jaw-dropping) by at least 3 s (Saal et al., 2017), suggesting that cardiac arrest, and the attendant collapse in cerebral perfusion, plays a key role in triggering unconsciousness.

Though a flat EEG is ultimately associated with the loss of consciousness in all its dimensions, the dynamic relations between the level of consciousness and the changes in cortical activity during a syncope is much less clear. At the onset of syncope, the EEG activity first exhibits diffuse and generalized abnormalities, characterized by the predominance of either theta, delta, or background suppression patterns (Ammirati et al., 1998; Sheldon et al., 1998; Wieling et al., 2009; Ziller and Natola, 2011; Figures 3A,B). While low-frequency EEG waves are most common at the time of fainting, they are not sufficient to explain unconsciousness, as conscious processes can be preserved during the slow phase of cortical activity associated with the syndrome of Cheyne-Stokes respiration (Karp et al., 1961). Further investigations will be required to determine how anoxia induces perturbations in brain connectivity and/or neural dynamics, which characterize the states of unconsciousness (Mashour and Hudetz, 2018).

### 6.2. Near-death experience

NDE is an extraordinary state of consciousness reported by part of survivors of life-threatening situations, such as cardiac arrest (10–25%), brain trauma (3%), major surgery, or coma (van Lommel et al., 2001; Greyson, 2003; Hou et al., 2013). Although each NDE is an idiosyncratic phenomenon, they all have in common a vivid and most often emotionally positive “core experience.” This includes a deep feeling of peacefulness, a sensation of entering a shiny tunnel, of revisiting their life in an instant, an out-of-body experience, or the strange feeling of an absence of a physical body (Greyson, 1983; van Lommel et al., 2001; Peinkhofer et al., 2019). NDEs are no longer considered as false memories or post-dramatic delusional episodes, but as genuine resurgence of a conscious activity that precedes the definitive collapse of mental life. Elucidating their neurobiological mechanisms provides one of the more fascinating challenges of clinical neurophysiology (Peinkhofer et al., 2019), even if a frequent spiritualist attitude tries to refute the possibility of a neuronal explanation (van Lommel, 2013).

Data obtained from animal models and electroclinical investigations in patients undergoing withdrawal care due to extensive systemic critical illness have led to the hypothesis that NDEs are subtended by the anoxia/ischemia-induced surge of high-frequency cortical activity. Borjigin et al. (2013) were the first to identify a transient burst of highly coherent gamma oscillations in the rat neocortex within the first 30 s following cardiac arrest. They concluded that the mammalian brain at near-death generates a cortical activity similar to that encountered during heightened consciousness, a process that could therefore explain NDEs. This hypothesis was further supported by measurements of the bispectral index (BIS)—combining EEG and EMG information—after cessation of life support in human patients with a terminal illness. Chawla et al. (2009, 2017) found that the loss of blood pressure was followed by a single, transient (~ 5 min. duration), spike in BIS values that approached levels normally associated with consciousness. A perimortem increase in the EEG gamma-band was also observed in patients during care withdrawal in absence of any hypnotic or anesthetic drugs (Auyong et al., 2010), indicating that the conscious-like type of cortical activity is a genuine response of the dying brain and not a drug-related artifact. More recently, EEG recordings from a patient undergoing cardiac arrest after subdural hematoma, revealed modulation of gamma activity by alpha and theta rhythms after cessation of cerebral blood flow, suggesting that the human brain may possess the ability to generate coordinated activity during the near-death period (Vicente et al., 2022).

The “high-frequency hypothesis” of NDEs is however highly speculative. First, the precise timing of NDEs remains unknown and it is therefore premature at this time to assert their neurophysiological correlates. Secondly, this hypothesis does not take into account the vividness, hallucinatory and hedonic aspects of the reported sensations. Because NDEs share similarities with altered states of consciousness induced by psychoactive drugs, such as ketamine, opioids, and some serotonergic psychedelics (Martial et al., 2019), it is conceivable to assume that the surge in cortical gamma activity, which may cause a consciousness-like experience, is accompanied by an abnormal and widespread release of various brain amines in the forebrain, resulting in the typical NDE phenomenology.

## 7. The twilight-zones model

The cortical modifications accompanying dying are globally unchanged over the various mammal species examined, and similar in the case of global cerebral ischemia/anoxia (cardiac arrest or brain arteries occlusion), asphyxia, or drug-induced respiratory arrest. They are subtended, regardless of the cause of death and the variety of phenomena, by the fast decline in cell metabolism due to oxygen deprivation. Cortical dynamics as death approaches are due to insurmountable pathogenic mechanisms, as their temporal profile is independent of preexisting endogenous brain activities, and they are not significantly affected by anesthetics. Furthermore, although the experimental data presented here were obtained primarily in humans and rodents, it is likely that the pattern of extinction of cortical activities and functions during death is similar in all mammals since it results from a common neuronal response to oxygen deprivation within this phylum. The new model of death described below is thus probably generalizable to all mammalian species, self-determining, and not related to a specific pre-mortem brain state.

**Figure 6:**
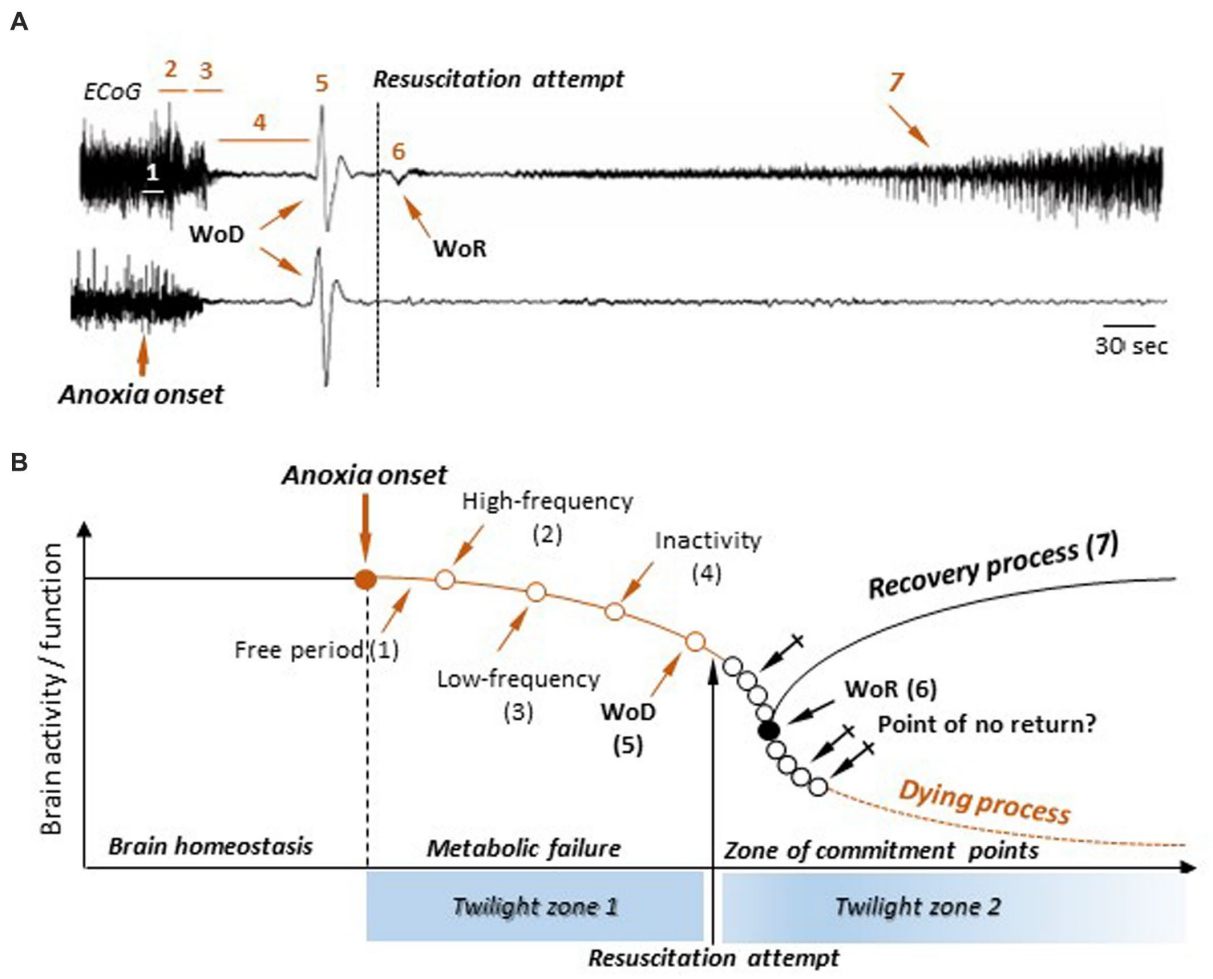
A new process-event model of death and resuscitation and its attendant *twilight zones*. **(A)** ECoG recordings in rats during the normoxic state, the anoxic period, and during a successful (top) or failed (bottom) resuscitation attempt (modified from Schramm et al., 2020). The successive epochs and events in cortical activities are indicated: free period (1), surge of high-frequency pattern (2), burst of slow waves (3), electrical inactivity (4), the wave of death (WoD) (5), the wave of resuscitation (WoR) (6), and the return of cortical activities (7) (see text for detailed description). No significant differences could be detected in the first four sequences, as well as in the WoD properties, whether resuscitation was successful or not. **(B)** A new model of death and resuscitation incorporating brain *twilight zones*. The lack of oxygenation (anoxia) causes a metabolic failure and a breakdown in brain homeostasis, leading to a slow decline in neuronal activity and functions following the successive steps as indicated in **(A)**. This constitutes the first *twilight zone*, in which the brain evolves in a potential death process. In absence of return of brain oxygenation, brain enters a commitment zone, where the point of return to death occurs at an unidentified moment (crossed arrows). The second *twilight zone* occurs when a resuscitation attempt is made. If successful, the appearance of the WoR provides a marker of resumed cortical activity. Alternatively, during a failed resuscitation procedure, the WoR does not arise and the brain enters an irremediable dying process. However, this does not mean that the point of no return is detectable.

It appears now obvious that the gradual breakdown of cortical activities in response to persistent anoxia makes the classical event-model of death obsolete, as it postulates a sudden transition between life and death (Figure 1). This model is refuted by the plethora of neurophysiological data described here and must therefore be replaced by a new, composite, model including both state transitions and slow neural processes. As illustrated in Figure 6A (top) and Figure 6B, the new scenario of cerebral dying is based on the stereotypical sequence of brain electrical activity—as recorded with cortical EEG electrodes—consisting of (1) a latent period, during which no significant modifications are detected; (2) a transient surge of high-frequency waveforms followed by, (3) a low-frequency pattern that slows down and attenuates over time, (4) the establishment of an isoelectric brain state and, (5) the occurrence of a massive cellular AD, monitored as a large-amplitude slow wave in the EEG (WoD). As demonstrated by Schramm et al. (2020), the WoD is not a terminal brain phenomenon since a timely restoration of oxygen supply can restore cellular homeostasis, signaled by the appearance of the WoR (6). The WoR instigates the recovery of neuronal properties and the resumption of spontaneous cortical activities (7).

Remarkably, and it is one of the main novelties of the new death model, recovery of brain activity upon resuscitation attempt is not yet predictable. Although it is empirically recommended to stop resuscitative efforts in patients not responding to 20 min of advanced life support (Bailey et al., 2000), the minimal duration of global cerebral anoxia/ischemia that ineluctably leads to irreversible brain damage, and subsequent death, remains unknown. It is thus not currently possible to delineate a strict time window for a certainly successful, or certainly hopeless, resuscitation attempt. Furthermore, the early dynamical changes in cortical activities are apparently similar for both failed and successful resuscitation attempts (Schramm et al., 2020; Figure 6A, bottom vs. top), indicating that no real-time electrophysiological signature of impending death is presently available. The lack of near-death markers implies that a durable anoxia makes the brain enter *twilight zones* in which its functional outcome is uncertain and where no predictive sign of certain death is objectively measurable; the WoR being the sole indicator—at least in the rodents—of the resumption of spontaneous cortical activity.

Based on this knowledge, a mixed, process-event, model can be proposed (Figure 6B). Here, the alteration of ongoing brain activity by anoxia follows the previously described sequence of cortical changes, going from a high-frequency activity pattern to the WoD (Figure 6A, top). This constitutes the first *twilight zone* (Figure 6B), during which neuronal activity and function gradually decline due to metabolic failure. Alertness and of any form of conscious experience are probably lost during this period. While the exact timing of consciousness loss remains unknown, it likely comes before the complete suppression of spontaneous activity (isoelectric state). It is therefore likely that the “death of consciousness” (irreversible unconsciousness) occurs while there is still spontaneous electrical activity in the brain, even if it is residual, and the cortical neurons are still able to process information, albeit in a degraded manner. Complete cortical function failure is achieved at the time of WoD, as collective ADs in the cerebral cortex suppress both integrative functions and excitability in neurons and cortical networks. From this point on, the brain enters a second *twilight zone* (Figure 6B), where its outcome becomes uncertain again. No commitment point toward a dying process (Point of no return) is currently identifiable after the WoD, even if they do occur, but at indeterminate times (crossed arrows). In this model, a resuscitation attempt generates a bifurcation point, with various possible timings (Figure 6B, empty black circles), from which the brain is either definitively engaged in a dying process or initiates a recovery process. In the latter case, the commitment point is signaled by the WoR, since it inaugurates the recovery of a normal neuronal polarization and the restoration of spontaneous cortical activity, but without prejudging possible disabling brain sequelae.

## 8. Conclusion

Human death is the death of the human brain. Understanding death, therefore, requires understanding the mechanisms subtending the definitive dissolution of brain activities and functions. Based on clinical and experimental results, death can no longer be viewed as an event or a long-drawn-out process. It is a time-dependent cascade of multifaceted neuronal changes, ranging from progressive attenuation in spontaneous synaptic patterns to the emergence of a discrete event represented by a sudden shift in neuronal electrical potential and cell size. The complete death sequence lasts a few minutes and none of the specific brain alterations alone constitute a point of no return to irreversible brain damage or death. The recent discovery of a post-anoxic resuscitation wave in rodents, heralding the resumption of cortical activity, provides the premise for a non-invasive, real-time, marker for a positive prognosis of patients with global cerebral ischemia. However, no brain event, or delineated neuronal process, is yet available to predict impending death. The demarcation between life and death resembles rather a neurophysiological horizon, the borders of which will be elucidated by examining the neuronal correlates of the *twilight zones* of a brain suddenly devoid of metabolism. Based on this new knowledge, we could finally unveil the specific neuronal and/or neural networks mechanisms underlying the long-term deleterious consequences of global cerebral anoxia or the positive neurological outcome after an attempt of resuscitation.

## Author contributions

SC conceived and wrote the manuscript.

## Funding

Part of the research described in this paper was supported by the “Investissement d’Avenir” program ANR-10-IAIHU-06, and Sorbonne University (Emergence-2019).

## Conflict of interest

The author declares that the research was conducted in the absence of any commercial or financial relationships that could be construed as a potential conflict of interest.

## Publisher’s note

All claims expressed in this article are solely those of the authors and do not necessarily represent those of their affiliated organizations, or those of the publisher, the editors and the reviewers. Any product that may be evaluated in this article, or claim that may be made by its manufacturer, is not guaranteed or endorsed by the publisher.
